# Morphology of palatally displaced canines and adjacent teeth, a 3-D evaluation from cone-beam computed tomographic images

**DOI:** 10.1186/s12903-018-0617-0

**Published:** 2018-09-04

**Authors:** Rosalia Leonardi, Simone Muraglie, Salvatore Crimi, Marco Pirroni, Giuseppe Musumeci, Rosario Perrotta

**Affiliations:** 1 Department of Orthodontics, Policlinico Universitario “Vittorio Emanuele”, University of Catania, Catania, Italy; 2 Department of Maxillofacial Surgery Policlinico G Martino, University of Messina, Messina, Italy; 3 Private practice, Bologna, Italy; 4 Department of Biomedical and Biotechnological Sciences, Human Anatomy and Histology Section, School of Medicine, University of Catania, Catania, Italy; 5 Department of Plastic Surgery, Director of the Master’s Degree in Plastic Surgery, University of Catania, Catania, Italy

**Keywords:** Cone-beam computed tomography, Surface-to-surface matching, Digital dentistry, Palatally displaced canine, Teeth morphologhy

## Abstract

**Background:**

The goal of this study was to investigate in patients with unilateral palatally displaced canine (PDC) the morphology of maxillary teeth from cone-beam computed tomography (CBCT) scans both on the PDC side and non-PDC side using a “surface matching” technique.

**Methods:**

CBCT images from 28 patients (mean age 16.04 ± 1.77 years) with unilateral PDC were selected. Each tooth in this study was segmented and then rendered into a 3D model using Mimics Research software and the root length was measured. Afterwards, 3D deviation analysis between the PDC and non-PDC side was carried out using Geomagic Control X software.

**Results:**

Statistically significant differences (*p* ≤ 0.001) were obtained when comparing the root lengths and volumes of lateral incisors from the PDC side, non-PDC side and control group. In accordance with the findings of 3D deviation analysis, statistically significant differences between the patients and control group were obtained for the lateral incisors and canines (*p* ≤ 0.0001) and greater differences were found for the tooth crowns and root tips.

**Conclusions:**

Lateral incisors adjacent to PDCs have shorter roots than contra-lateral lateral incisors. Furthermore, there were morphological differences between lateral incisors and canines in subjects with unilateral PDCs.

## Background

Maxillary canine impaction occurs in the general population with a reported prevalence ranging from 0.27 to 2.4%, depending on the population [1–3]. This condition affects female patients 2 to 3 times more frequently than males [4].

Although numerous possible factors are under assessment, it is certain that buccally displaced canines (BDC) and palatally displaced canines (PDC) are characterized by different etiopathogeneses [4–6]. Whilst, BDC is thought to be one result of insufficient space in the maxilla for the eruption of the maxillary canine, the etiology of PDC is still unclear and varied reasons have been postulated [4, 6, 7].

Besides the several causes of PDCs, the most debated opinions of respected researchers are the genetic theory [6, 7] and the guidance theory [4, 8]. Nonetheless, both theories agree on the important role played by the adjacent lateral incisor, as normal canine eruption is guided by the lateral incisor root of sufficient length (guidance theory), whereas small or peg-shaped lateral incisors are associated genetically to PDC (genetic theory).

Several studies of dental casts have already described a higher risk of PDCs in patients with tooth crown size reduction [9–13]. Furthermore, recently, two more studies utilizing cone-beam computed tomography (CBCT) [14] and multi-slice spiral computed tomography (CT) [15], demonstrated that the lateral incisors adjacent to PDCs are smaller and the roots are shorter compared to those adjacent to normal canines. Another study [16] evaluating the crown-root angulation of the lateral incisor adjacent to PDCs on panoramic images, indicated that its root was angled more mesially compared to the lateral incisor adjacent to the normally erupted canine. So, according to this latter finding, the roots of lateral incisors contiguous to PDCs seem to show a deviation in form. However, as the authors stated themselves [16], their study has some limitations, in that measurements from panoramic X-rays tend to overestimate the mesial angulation of lateral incisors compared to a three-dimensional image, such as CBCT, thereby revealing an inherent error in using a two dimensional image to depict three-dimensional structures.

Therefore, although there is no consensus about the exact etiology of palatally impacted canines, it appears that the adjacent lateral incisor highlights an important role, either because its eruption and size are controlled by the same genes that control the eruption of the canine (genetic theory) or because its position in the arch influences the canine’s eruption path [16].

Recent advances in computer technologies and the rapid growth in the use of 3D imaging techniques provide more accurate evaluations and comparisons of anatomical structures. So, 3D ‘surface-to-surface’ matching of maxillary teeth from CBCT-derived models, could make morphological differences observable between homologous teeth from the two semi-arches as well as providing precise measurements of tooth sizes (volumes, widths and heights).

As no study, to our knowledge, has evaluated maxillary central and lateral incisors as well as first premolars adjacent to PDCs with the surface-volume matching technique, the aims of this study were to investigate in patients with unilateral PDC:

- the dimensions and morphologies of the central and lateral maxillary incisors, canines and first premolars from CBCT images both on the PDC side and non-PDC side;
- to do ‘surface point-to-point’ matching of teeth on the PDC side and non-PDC side (canines with a normal eruptive patterns);
- to compare these findings with a control sample of patients with no PDC.

The null hypothesis of this study was that there are no differences in either tooth morphologies or sizes on the PDC side and non-PDC side.

## Methods

To determine the sample size, a power analysis was carried out (DSS Research, Washington, USA) which indicated that data from 18 participants would yield a confidence level of 95% and a Beta error level of 25%, making it sufficient to determine statistically significant differences.

So, the study group (SG) consisted of CBCT images from 28 consecutive patients (12 boys and 16 girls) who had been referred (between January 2016 and July 2017) to a private X-ray practice specialising in CBCT from large record pools. The scans were de-identified to protect patient confidentiality and ethic approval was obtained from the Ethic Committee of Policlinico Vittorio Emanuele, Catania, (reference number #4217). Also, a written informed consent was obtained from all patients included in this study. All procedures performed in this study involving human participants were in accordance with the ethical standards with the 1964 Helsinki declaration and its later amendments or comparable ethical standards.

The inclusion criteria were: good quality scan, sufficient field of view (FOV) for including the entirety of maxillary teeth, and the presence of a unilateral maxillary canine impaction.

The exclusion criteria were: movement artifacts, patients affected by cleft palate, dentofacial deformities, teeth anomalies (except for the PDC) or agenesis, caries, fillings, restorations, and conspicuous abrasions on the cusps and edges, buccal or midalveolar impacted canines, severe root resorption and teeth with dilacerated roots.

The mean age of the patients at the time of the CBCT scans was 16.04 ± 1.77 years.

These patients were age-and-gender matched with 25 subjects (11 boys and 14 girls, mean age??), affected by third molar impaction, who served as the control group (CG). The inclusion and exclusion criteria were the same as the SG plus the absence of PDC.

All CBCT images were taken with the NewTom 3G (QR SRL, Verona, Italy) device (110 kV, 6.19 mAs, 0.25 mm voxel size, and 8-mm aluminum filtration) with the patient in maximum intercuspation and Frankfort horizontal plane parallel to the floor following common CBCT imaging protocols [17].

All the data sets were exported and converted using the Digital Imaging and Communications in Medicine (DICOM).

To obtain 3D surface mesh models of the teeth and measure the root lengths of every single tooth (central incisor, later incisor, canine and first premolar of the maxillary arch), the DICOM-formatted images were volume rendered with Mimics (Mimics Research, version 19.0.0.347, Materialise NV, Liege, Belgium). Surface mesh models were further analyzed with reverse engineering software (Geomagic Control X, version 2017.0.0, 3D Systems, USA) to calculate the total volume of every single tooth model and to achieve a point-to-point surface analysis between the 3D models of the teeth on the PDC and non-PDC sides. The scanning, segmentation, and model fabrication protocols used in this study were previously validated and described [17–19].

**Fig. 1:**
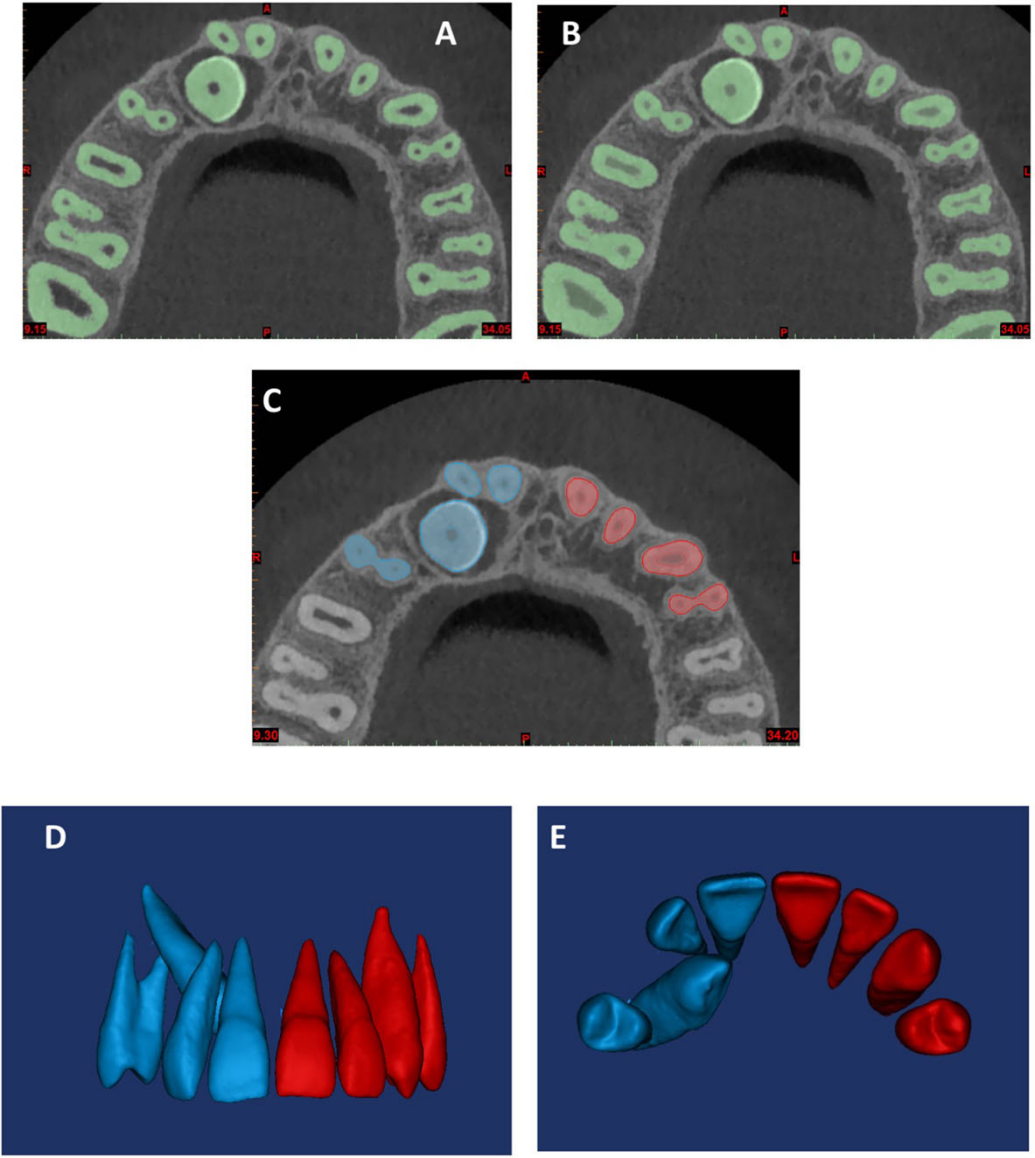
Generation of the segmentation mask (**a**, **b**) using the ‘automatic threshold’ function of the software; pure segmentation masks (**c**); 3D surface models of the teeth (**d**, **e**)

Briefly, the work-flow to obtain root lengths, volumes and surface-to-surface matching is described below in seven steps:

Step 1- Generating the segmentation mask: to develop the segmentation mask (Fig. 1a), we used the ‘automatic threshold’ function of the Mimics Research software. The threshold was adjusted scan by scan to automatically detect the Hounsfield values and boundaries of all the teeth. During generation, the pulpal tissue of each tooth was included in the volumes to minimize the errors in discriminating dentin from pulpal tissue as an added source of variation (Fig. 1b). Then, The selected mask was then cropped into eight segments providing axial, sagittal and frontal views, by using the ‘Crop Mask’ function of the software, to obtain pure segmentation masks of every single tooth (Fig. 1c). Thereafter, the quality and precision of the single tooth mask was improved first by manually erasing, slice by slice, in the sagittal and axial views, the excess parts of the segmentation mask outside the tooth contour: for example the parts of the other teeth included in the mask during the cropping process. Later, the mask was smoothed and finely adjusted by using the interactive ‘Contour Edit’ function, to improve quality and contour delineation. To avoid errors during the procedures, the PDC-side masks were colored blue while the non-PDC-side tooth masks were colored red.

Step 2- Segmentation: After generating the segmentation mask for each single tooth, to obtain the three-dimensional surface models of the teeth in this study, each mask was rendered into a three-dimensional model using the ‘3D calculation’ function (Fig. 1d-e).

**Fig. 2:**
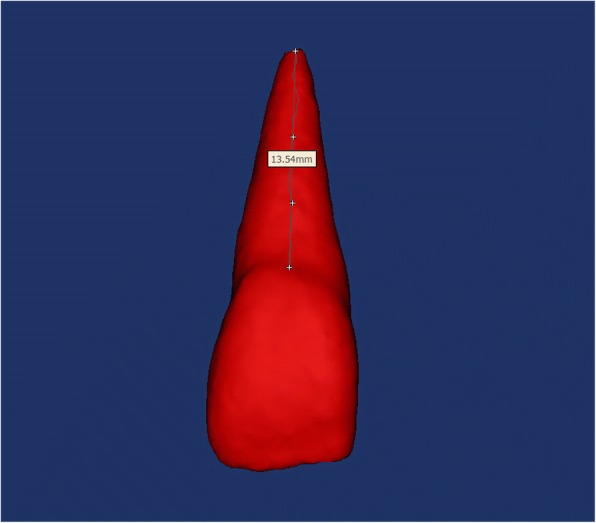
The root lengths of each tooth were measured by selecting 4 points: the most apical point of the CEJ, two points respectively 4 mm and 8 mm apical to the CEJ level and a point at the apical foramen

Step 3- Measurements: the root length was measured for each tooth, (i.e. the surface distance between the labial cement enamel junction and the root apex) (Figs. 2 and 5).

The measurements were made with the Mimics measurement tools, with a precision of 0.1 mm, directly on the rendered digital tooth models. The images were magnified by 300% to facilitate better visuals and to avoid errors during the point selections. The image was reoriented with the buccal surface of the tooth crown facing out of the computer screen, and the tooth standing vertically in the coronal and sagittal views. Then, using the surface measuring tool of the Mimics software, 4 points were identified on the buccal surface of the tooth root. These points were: the most apical point of the CEJ, two points respectively 4 mm and 8 mm apical to the CEJ level and a point at the apical foramen level. Each point was selected at the horizontal midpoint of the root surface. (Fig. 2).

**Fig. 3:**
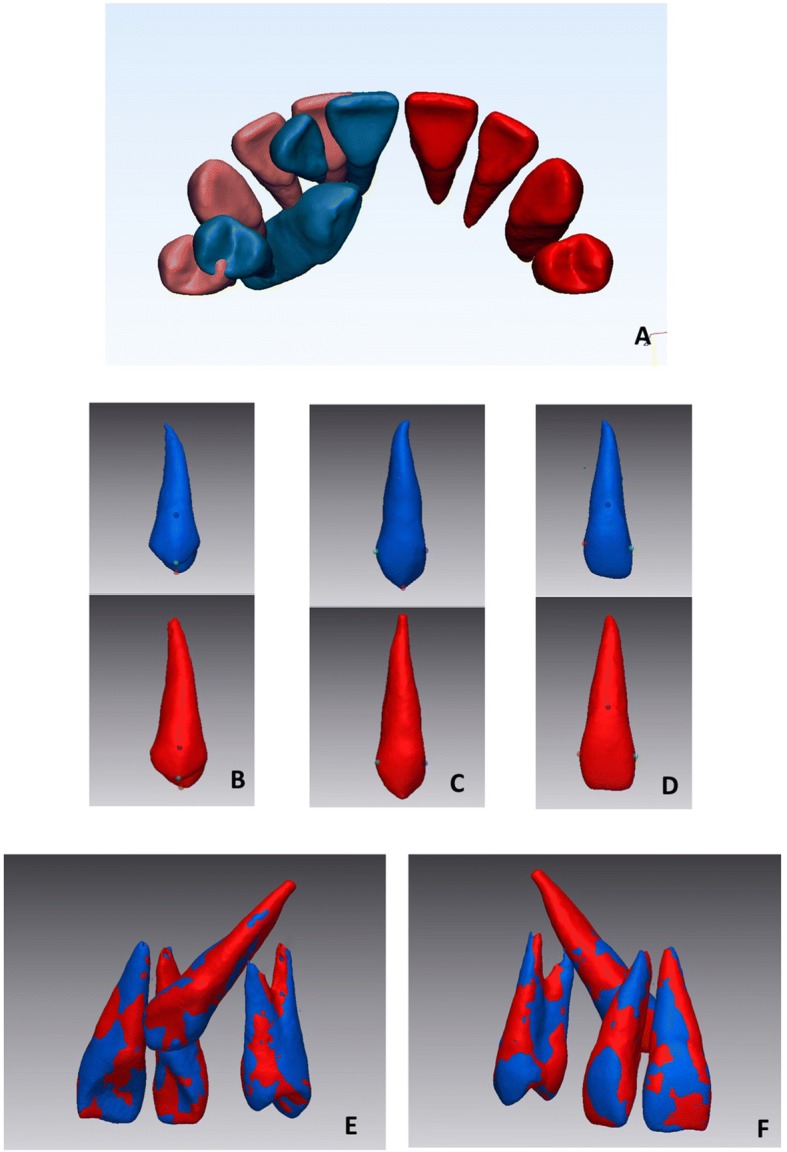
3D tooth models (blue PDC side, red normal side) (**a**); selection of 3 points on the surface of the specular tooth models (**b**, **c**, **d**) (see text for points) for the first alignment; second alignment using the ‘best fit’ function of the software, palatal view (**e**) and vestibular view (**f**)

Step 4- Mirroring: The 3D tooth models were exported to Geomagic Control X software as a stereo-lithographic format file (.stl) and the non-PDC side teeth were mirrored by converting their image orientation (Fig. 3a).

Step 5- First registration, point-based: a manual point-based superimposition selected the same 3 points on the surface of the specular tooth models to align the mirrored non-PDC side teeth models with the PDC-side teeth models. These points were the buccal and palatinal cusp tips and the deepest vestibular point at the cement enamel junction (CEJ) level, for the first premolar (Fig. 3b), the cusp tip and the mesial and distal points on the largest diameter of the crown for the canine (Fig. 3c); the deepest vestibular point at the CEJ level and the mesial and distal points on the largest diameter of the crown for the permanent incisive (Fig. 3d).

Step 6- Final registration: to enhance superimposition quality, a final registration was performed using the ‘Best fit alignment’ option in the Geomagic Control X software. The reference data-set was obtained, setting the precision of the registration to at least 0.2 mm (tolerance type: ‘3D Deviation’) and the percentage of surface registration polygons to the maximum 100% (Fig. 3 e-f).

**Fig. 4:**
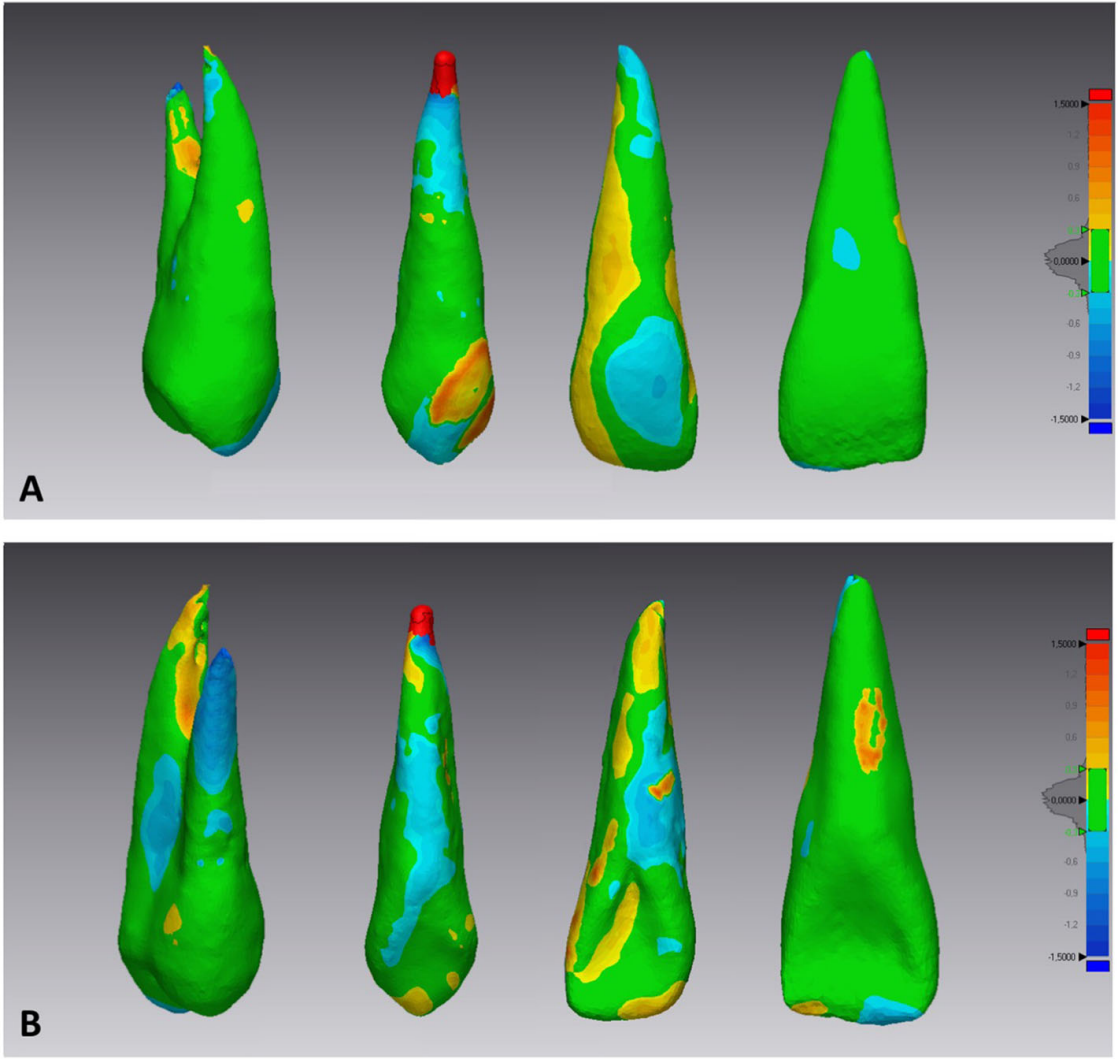
Deviation analysis between the specular tooth models from the PDC side and non-PDC side. The colored map shows the deviations (negative blue, positive red) between the mesh models. **a**) vestibular view, **b**) palatal view

Step 7- 3D Deviation analysis: after superimposition, 3D surface deviation analysis was carried out with the Geomagic Control X software automatically calculating the means and maximum values of the distances between the specular 3D crown models, measured between 100% of the surface mesh points, and representing them on a color analysis map. These values were visually displayed on a color map which showed the deviation in different colors (blue for maximum negative, red for maximum positive, green for the range tolerance). Distances greater than 0.3 mm are represented in red or blue while distances within the tolerance range (+ 0.3 to − 0.3 mm) are represented in green (Fig. 4 a-b). This map shows the surface distance (Euclidean distance) distributions between the entirety of the segmented tooth’s surface points on the PDC side and its corresponding segmented tooth points on the non-PDC side.

The maximum deviation was set to 1.5 mm.

After the deviation analysis, the percentage (%) was calculated for all the deviation values within the tolerance range (− 0.3 to + 0.3 mm). These values indicated the matching percentage between the pairs of corresponding tooth models.

To minimize random and systematic errors, all the digital measurements on CBCT images were performed by a single examiner, with 25 years of orthodontic experience (R.L.). The examiner analyzed only 5 CBCT images each day to avoid fatigue. The CBCT images were examined in blind sequence.

To determine intra-observer error, 20 CBCT images were randomly selected and all the measurements repeated 6 weeks after the first examination by the same specialized operator with no knowledge of the first measurements.

### Statistical analysis

All measurements were recorded on Microsoft Excel® spreadsheet (Microsoft, Redmond, WA, USA) and analyzed using SPSS® version 24 Statistics software (IBM Corporation, 1 New Orchard Road, Armonk, New York, USA).

Intra-examiner reliability was assessed using Dahlberg’s formula [20] (method error = √Σd_2_/2n, where d is the z difference between the two measurements of a pair, and n is the number of samples).

The Kolmogorov–Smirnov test was used to test the normality of the data. As all the data was normally distributed with homogeneous variance, parametric tests were used to evaluate the volumetric and linear data from the PDC side and non-PDC side. A paired *t*-Test was used to compare the root lengths and volumes of teeth from the PDC side and non-PDC side. Measurements of the PDC side, non-PDC side and control group were further analyzed by one-way Analysis of Variance (ANOVA) to evaluate if they were statistically significant to accept or reject the null hypothesis. The mesh percentages from the PDC sample and control sample were compared by *t-*test.

The significance level was set at *p* ≤ 0.05. *p* values less than 0.05 were considered statistically significant.

## Results

Of the 28 patients with unilaterally impacted canines, 13 were on the right side and 15 on the left. The intra-examiner reliability of the measurements showed a high correlation with Dahlberg’s values not greater than 0.99 (*p* < 0.000) for both the volumetric and linear measurements.

**Fig. 5:**
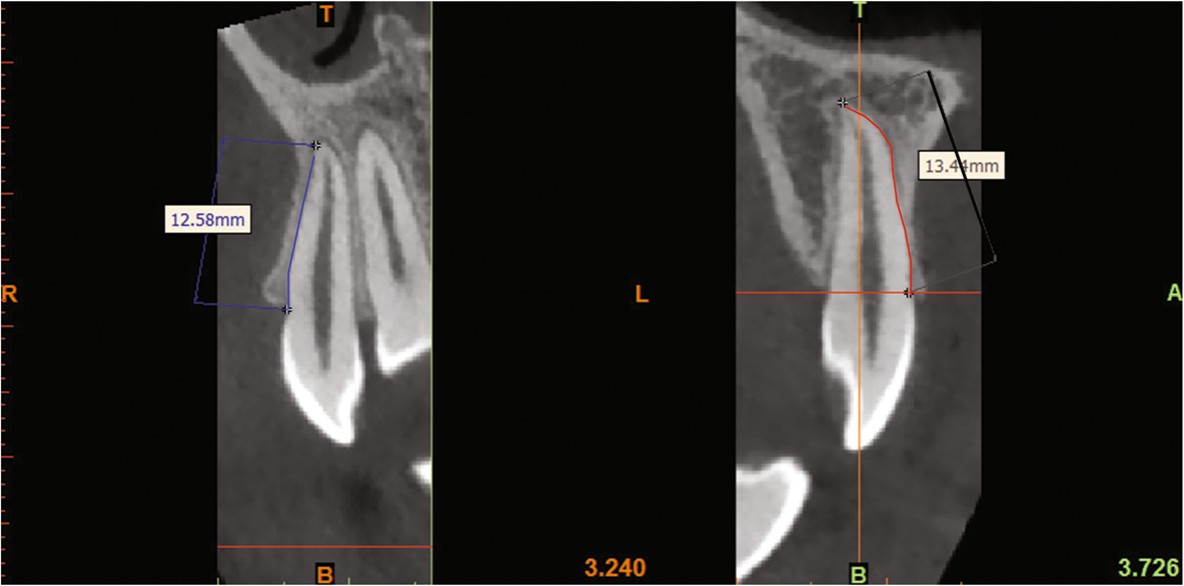
CBCT scan of an upper lateral incisor on the palatally displaced canine side (Right) and of an upper lateral incisor on the non-palatally displaced canine side (Left). Measurements of the root lengths in millimeters at root surfaces from the labial cement enamel junction to the root apex

The descriptive statistics for the root lengths (mm) and volumes (mm^3^) of subjects with palatally displaced canines (PDC side and non-PDC side) and controls are shown in Table 1. Lateral incisors adjacent to PDCs showed a mean root length of 10.43 ± 0.72 mm, and this being shorter than that of the lateral incisor from the non-PDC side of the same patient (11.43 ± 0.78 mm), and from the control group (10.71 ± 0.86 mm). On average, lateral incisors adjacent to PDCs were shorter by 1 mm when compared to those on the non-PDC side of the same subject, and were 0.48 mm shorter compared to controls, these differences being statistically significant (*P* ≤ 0.001), (Fig. 5). Even though there were some differences in tooth root lengths were obtained for the other teeth, they were only statistically significant for the first premolar (*P* ≤ 0.05) which was shorter than those of controls.

**Table 1:** Mean Values and standard deviations (±). Comparison between PDC side, non-PDC side and control group for both radicular length (mm) and volume (mm^3^)

		PDC		Non-PDC		*P* value	Control		*P* value
Root length	Central incisor	11.72	±0.67	11.71	±0.71	NS	11.63	±0.73	NS
	Lateral incisor	10.43	±0.72	11.43	±0.78	*	10.71	±0.86	†
	Canine	14.88	±1.37	14.84	±1.16	NS	14.63	±0.82	NS
	First premolar	11.07	±0.95	11.19	±0.95	NS	10.46	±0.64	*
Volume	Central incisor	402.23	±27.90	403.72	±24.34	NS	399.38	±21.03	NS
	Lateral incisor	308.26	±35.68	361.43	±31.41	*	351.26	±12.13	‡
	Canine	558.97	±49.30	553.68	±38.63	NS	544.47	±36.36	NS
	First premolar	383.26	±32.01	390.54	±29.92	NS	371.05	±27.56	NS

*P* value based on one-way ANOVA. *NS* non significant; *P ≤ 0.05; † *P* ≤ 0.001; ‡ *P* ≤ 0.0001

As for volume, the most noticeable finding was that lateral incisors from the PDC side were smaller (308.26mm^3^) compared to the non-PDC side (361,43 mm^3^) and these differences being statistically significant (*P* ≤ 0.0001) (Table 1).

The descriptive statistics and *P* values of the mesh matching percentages of subjects with unilateral PDCs versus controls are shown in Table 2. The percentage of mesh matching according to the ‘surface-to-surface’ analysis for the control group ranged from 84.75 for the first premolar to 85.37 for the central incisor, whilst for the PDC group from 71.48 for the lateral incisor to 84.13 for the central incisor. Lateral incisors and canines showed the lowest matching percentages of the upper teeth in subjects with unilateral PDCs compared to controls. That is to say that the percentage differences in mesh point matches for lateral incisors and canines in the study group were lower than controls, demonstrating that there are some morphological mismatches of these teeth in subjects with palatally displaced canines. Statistically significant differences between the two groups were obtained for the lateral incisors and canines (*P* ≤ 0.0001).

**Table 2:** Comparison between study group and control group for matching

		Mean and standard deviation (±)				
		Sample group		Control group		*P* value
Matching	Central incisor	84.13	±3.37	85.37	±2.52	NS
	Lateral incisor	72.48	±2.64	83.49	±2.02	*
	Canine	77.20	±2.27	83.21	±2.13	*
	First premolar	81.45	±3.14	82.75	±3.10	NS

*P* value based on independent *t-*test. *NS* non significant; *P ≤ 0.0001

Therefore, 3D tooth symmetry using deviation analysis corroborated that tooth morphology (crown and root) on the PDC side and in controls not only differed for lateral incisors but also for palatally displaced canines. Interestingly, these differences were more evident at the root tips and in most of the crowns, according to the color-coded map, where red and blue areas highlighted the higher deviations.

## Discussion

This investigation tested the assumption that upper tooth dimensions and morphologies for subjects with palatally displace canines differ from those of the teeth adjacent to normally erupted canines in the same subject and to those of controls. Our findings demonstrate, for the first time, that lateral incisor and displaced canine morphologies and dimensions on the PDC side differ from those on the non-PDC side and controls.

About 50 years ago, Miller [21] and Bass [22] independently observed that the prevalence of palatal displacement was greater when lateral incisors were congenitally missing. They concluded that the absence of the lateral incisor denied the canine its guidance, permitting it to migrate palatally. These conclusions, were based on clinical impressions from viewing a number of patients in the clinic and not from a disciplined study of a large sample of affected patients vs an appropriate random control group.

Currently, the two most popular theories reported in the literature that have gained some degree of consensus worldwide, are the guidance theory [8, 22–25] and the genetic theory [4, 8, 9, 23, 26–29], which both share the belief that certain genetic features occur in association with the cause of palatal displacement of the maxillary canine. However, insofar there is no single and exclusive cause [30].

The results from this investigation seem to support shorter root lengths and reduced volumes of the upper lateral incisors involved in PDC as it can exert a powerful local influence.

Our results corroborate previous findings on lateral incisor root length [14, 15] being shorter on the PDC side. Additionally, for the first time a deviation in 3D tooth morphology of lateral incisors and canines of subjects with unilateral palatally displaced canines was demonstrated, as established by reverse engineering. Regarding lateral incisor root length, the results of our study demonstrated that its root was shorter on average by 1.00 mm compared to the contra-lateral side of the upper jaw, and by 0.48 mm compared to the lateral incisor root lengths of controls. Our findings substantiate previous studies [14, 15] which highlighted that lateral incisors had shorter mean length ranging from 0.78 mm [15] to 2.1 mm [14] and a smaller crown volume [15] on the PDC side.

By contrast, there were no statistically significant differences in root lengths or volumes for the maxillary central incisors, canines and first premolars (except for the length of the first premolar) between the three groups.

As far as volume is concerned, the lateral incisor on the palatally displaced canine side, showed a statistically significant smaller volume when compared to the lateral incisor on the non-displaced canine side and to lateral incisors from the control sample. However these findings are hardly comparable to the only previous study [15] because that investigation’s data referred only to crowns and not to entire teeth.

The most noticeable and evident findings of our study concern the difference in surface-to-surface matching of upper teeth obtained from PDC patients and those of the control group. For the first time, a mesh analysis is presented, i.e. a 3D surface–to-surface matching from the affected and unaffected sides of PDC patients which was compared to data from the control sample. Interestingly, upper lateral incisors and canines from the PDC group displayed a lower percentage of matching when compared to homologous teeth of the control sample. Since the tolerance range was set at − 1.5 to + 1.5 mm, it is improbable that these mis-matchings were due to extreme curvature of the lateral incisor and canine roots. In fact this study was designed only to detect subtle differences which can hardly be appreciated by visual assessment, so CBTC scans with extreme variation in tooth morphologies were not included in the sample. Indeed, several previous studies have well documented a significant association between tooth morphology (lateral incisors and displaced canines) and palatally displaced canine [10, 14, 15, 23], however none of these studies has reported morphological differences, of small entities between homologous teeth from the PDC side and non-PDC side. The 3D surface-to-surface matching technique which was used in our research can reveal any differences, even small, in morphology and tooth size.

According to our results it may be suggested that individuals with shorter maxillary lateral incisor roots and morphological differences in the lateral incisors and canines (crowns and roots) are vulnerable to impaired eruption of the canine. Furthermore, before beginning orthodontic treatment, clinicians should be aware of both shorter lateral incisor root lengths and lateral incisor and canine dimorphism both in crowns and roots.

There are some limitations to this study in that the palatally impacted canine group from the radiology practice did not represent the general population. In fact, there is a tendency for clinicians to only refer patients with more severely impacted canines or other complicated cases for cone-beam volumetric tomography. Another weakness of the study, that puts this investigation at a risk of bias, is that researcher was obviously not blinded, when generating the segmentation masks for PDC and non-PDC-side.

## Conclusions

The lateral incisors adjacent to palatally displaced canines have significantly shorter roots than contra-lateral lateral incisors adjacent to normally erupted canines. Furthermore, there are also differences in lateral incisor and canine morphologies in subjects with PDCs compared to controls.

Furthermore, this study provides new evidence that even the dimorphism of a small entity of permanent lateral incisor is involved in the canine palatal displacement process, besides the already described tooth anomalies.

## Abbreviations

3D: Three-dimensional
ANOVA: One-way Analysis of Variance
BDC: Buccally displaced canine
CBCT: Cone beam computed tomography
CEJ: Cement-enamel junction
CG: Control group
FOV: Field of view
PDC: Palatally displaced canine
SG: Study group
